# Evolution of periodicity in periodical cicadas

**DOI:** 10.1038/srep14094

**Published:** 2015-09-14

**Authors:** Hiromu Ito, Satoshi Kakishima, Takashi Uehara, Satoru Morita, Takuya Koyama, Teiji Sota, John R. Cooley, Jin Yoshimura

**Affiliations:** 1 Graduate School of Science and Technology, Shizuoka University, Hamamatsu, 432-8561, Japan; 2 Nagoya College, Toyoake, Aichi Pref., 470-1193, Japan; 3 Department of Mathematical and Systems Engineering, Shizuoka University, Hamamatsu, 432-8561, Japan; 4 Department of Zoology, Graduate School of Science, Kyoto University, Sakyo, Kyoto 606-8502, Japan; 5 Department of Ecology and Evolutionary Biology, University of Connecticut, Storrs, CT 06268-3043, USA; 6 Department of Environmental and Forest Biology, State University of New York College of Environmental Science and Forestry, Syracuse, NY 13210, USA; 7 Marine Biosystems Research Center, Chiba University, Uchiura, Kamogawa, Chiba 299-5502, Japan

## Abstract

Periodical cicadas (*Magicicada* spp.) in the USA are famous for their unique prime-numbered life cycles of 13 and 17 years and their nearly perfectly synchronized mass emergences. Because almost all known species of cicada are non-periodical, periodicity is assumed to be a derived state. A leading hypothesis for the evolution of periodicity in *Magicicada* implicates the decline in average temperature during glacial periods. During the evolution of periodicity, the determinant of maturation in ancestral cicadas is hypothesized to have switched from size dependence to time (period) dependence. The selection for the prime-numbered cycles should have taken place only after the fixation of periodicity. Here, we build an individual-based model of cicadas under conditions of climatic cooling to explore the fixation of periodicity. In our model, under cold environments, extremely long juvenile stages lead to extremely low adult densities, limiting mating opportunities and favouring the evolution of synchronized emergence. Our results indicate that these changes, which were triggered by glacial cooling, could have led to the fixation of periodicity in the non-periodical ancestors.

True periodicity, involving synchronized adult emergences, fixed life-cycle lengths, and intervals between emergences with no adults present, is rare among insects^1^. The periodical cicadas of eastern North America (*Magicicada* spp.) exhibit 17- and 13-year periodical life cycles, the most extreme periodical life cycles known in insects^2^,^3^,^4^,^5^,^6^. *Magicicada* are also characterized by mass emergences and division into regional populations that share emergence years. In periodical cicadas, each “emergence year-class” is specifically called a “brood”^2^,^3^. Cicadas (Order: Hemiptera) are singing insects that are widely distributed from the tropics to temperate zones. Almost all known cicada species have size (weight)-dependent maturation with variable-length life cycles. Their nymphs grow underground at a rate set by resource availability (water from roots that depends on the cumulative temperatures of trees), and when they reach a threshold maturation size, they emerge from the ground and moult into short-lived adults^7^,^8^.

In contrast, *Magicicada* exhibit time-dependent maturation and emerge after exactly 13 or 17 years. In the current climate (postglacial period), most of them appear to be able to grow to threshold maturation size well within their lifespans of 13 or 17 years^9^. However, some small, under-developed adults are occasionally found. This observation means that *Magicicada* emerge after a fixed length of time, irrespective of reaching a threshold body size. Thus, a prominent question regarding the evolution of periodical cicadas is why they transitioned from size-dependent to time-dependent maturation^10^,^11^.

A number of hypotheses address the evolution and maintenance of periodicity and mass emergence in *Magicicada*. One hypothesis is that periodicity with mass emergence is a strategy for predator avoidance^12^,^13^,^14^,^15^, but this hypothesis has a significant flaw: It cannot account for the rarity of periodicity. All cicadas face predation pressure, but only a small number of species are known to be periodical, e.g., one in India^16^,^17^ and another in Fiji^18^. Other hypotheses suggest that geological cooling during glacial periods triggered the evolution of periodical cicadas^19^,^20^, but these hypotheses are also limited because many species of cicadas live in glaciated areas, yet only a few are periodical. The unique combination of natural history traits in *Magicicada* argues for an explanation that divides the evolution of *Magicicada* into smaller problems.

One focus of *Magicicada* studies has been on the relationship between the mass emergence of these species and the unusual, prime-numbered life cycles of 13 or 17 years. However, the persistence of prime-numbered cycles has been shown to be a plausible outcome of circumstances in which already-periodical cicadas face disproportionately high predation pressures when they emerge at low densities^14^,^21^,^22^,^23^. Moreover, repeated instances of life-cycle switching from 13 to 17 years or vice versa over evolutionary time^24^ suggest that these life cycles are stable strategies, whereas other alternatives are not.

The history of periodical cicadas involves periods of confinement in forested refugia when other regions of the eastern half of North America were still directly or indirectly rendered unsuitable by glaciation. During ice ages, the emergence of any cicadas with size-dependent life cycles may have been delayed many years due to the reduction in yearly cumulative temperatures. It is possible that the long nymphal stage preceding such emergence delays could have significantly increased nymphal mortality, resulting in extremely low adult densities. Furthermore, populations may have been vulnerable to extinction due to several factors, including limited mating opportunities, severe predation pressure, and high mortality under marginal climate conditions^23^,^25^.

One way that the ancestors of *Magicicada* may have survived these challenges was by adopting periodical emergences. When geological cooling advances, average juvenile (nymphal) period elongates, and at the same time the variation in juvenile growth period increases, resulting to an extremely low adult density. First, consider the elongation of juvenile period as follows. Suppose the ancestral cicadas emerge every year and the growth period is about 7 years with 100 adult individuals emerging in a small habitat. This means a total of 700 (100 × 7 year) adults emerging in 7 years. Next, cooling arrives and doubles the growth period to 14 years. If we assume that the overall mortality per nymph is identical, 700 individuals emerging in 14 years means 50 adult individuals per year. However, this nymphal mortality should be incorrect. Otherwise, nymphal mortality per year is halved since the nymphal stage is doubled. Assuming the nymphal mortality per year is constant, the emerging adult population size becomes 25 individuals per year (total population size is 25 × 14 = 350 individuals). Thus, under the same mortality condition, the adult density per year should be quartered (i.e., 1/4). However, geological cooling should drastically increase the annual juvenile mortality due to freezing and desiccation. Thus the resulting yearly adult population size becomes exceptionally small compared with those before geological cooling.

Geological cooling also reduces the adult density by increasing the variation in juvenile growth rates. For example, the variation in yearly emergence should increase significantly when the average yearly emergence is 5 individuals compared with 100 individuals. Therefore, the adult emergence may become vacant in most years, resulting to periodical emergence even under size-dependent maturation. Moreover, when the population size at a given year reaches a critically low density, the emergence of adult individuals should become almost periodical and nearly synchronized, even under size-dependent maturation. In this stage, the variation in juvenile growth further boosts adult density because of the loss of adults emerging in a wrong (previous or next) year. For example, when non-periodical adults lay one hundred eggs, some eggs (say 10) grow faster, resulting in one-year early emergence; while some others (say 10) are slower, resulting in one-year late emergence. These early and late emergences (total 20 adults) can be lost without producing any offspring. Because of this, adult density is reduced by 80% in every generation. By switching to periodical emergence with a fixed period (complete synchronization), this loss of emergence disappears. We hypothesize that this advantage of periodical emergence over size-dependent maturation can lead to the evolution (fixation) of periodical genes, during cooling, so that the population is at a lower risk of extinction.

In this paper, we specifically demonstrate how declining average temperature, which directly affects growth rate, could promote the evolution of time-dependent maturation and periodicity from an ancestor with size-dependent maturation. Under climatic cooling conditions, our model suggests that size-dependent cicadas could be replaced by periodical cicadas with life cycles of various year lengths. In our model runs, cicadas that acquire periodicity establish only one brood when the Allee effects are moderately strong. Slight variations in conditions affect model outcomes: cooler temperatures promote the establishment of longer cycles, while warmer temperatures promote the establishment of shorter cycles. Our results provide insights concerning the initial conditions necessary to provide a selective advantage for periodicity in North American *Magicicada*.

## Model

### Individual-based model (IBM)

**Figure 1:**
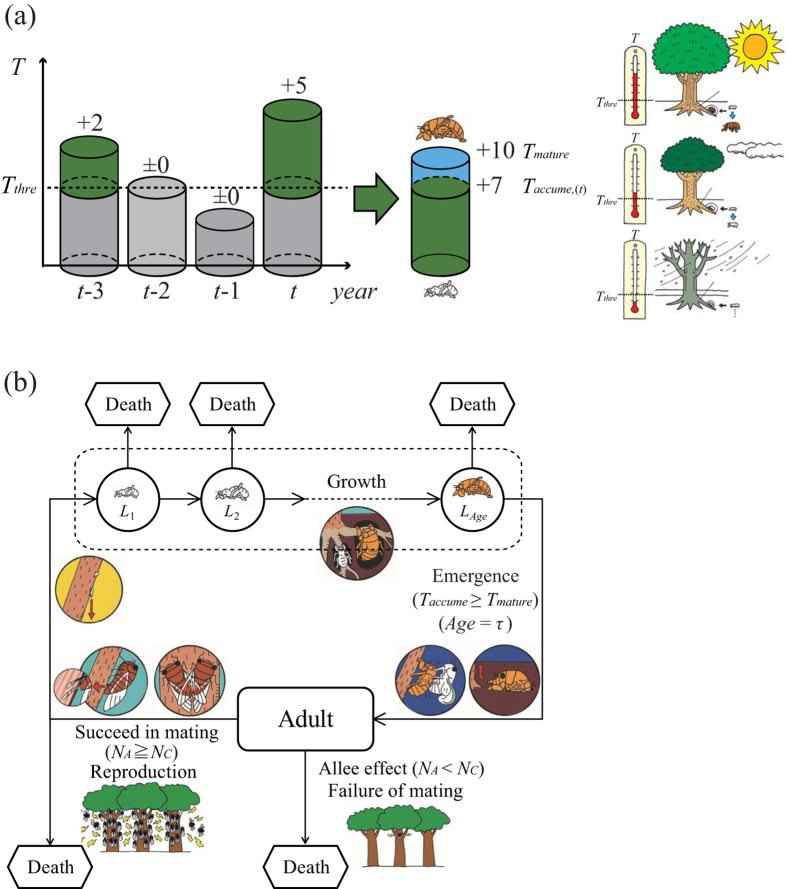
Illustration of the individual-based model (IBM). (**a**) Image of the accumulated temperature. The area in green above *T*_*thre*_ represents growth based on accumulated temperature. When the sum of accumulated temperature (*T*_*accume*_) reaches the threshold amount for maturation (*T*_*mature*_), the non-periodical cicadas emerge. (**b**) Simulation flow. The variable *T*_*thre*_ is defined as the temperature threshold of individual cicada *i*, such that if *T*_*t*_ ≤ *T*_*thre*,*i*_ (for some *t*), then *T*_*mature,i*_ is fixed, resulting in a longer time of emergence. An individual cicada nymph *i*(*t*) emerges at time *t* if *T*_*accume,i*(*t*−1)_ ≥ *T*_*mature*_. If *T*_*accume,i*(*t*−1)_ < *T*_*mature*_, a nymph cannot emerge the next year. Illustration by Yoshihiko Ishimori.

We build a simple individual-based model incorporating Mendelian inheritance and random mutation (Fig. 1, see supplementary information). We assume a one-locus, two-allele genetic system controlling emergence (i.e., determinant of maturation). In this locus, we assume two types of alleles: (1) temperature (size)-dependent alleles and (2) time-dependent alleles (i.e., temperature-independent or periodicity alleles) that have a specific lifespan length (e.g., 10–20 years). Alleles for periodicity of any given year cycle length are assumed to be dominant, whereas the temperature-dependent allele (ancestral allele) is recessive.

An average temperature (*T*_*mean*_, in an arbitrary unit) is set for each simulation with a value between 17.0 and 20.0 (0.1 intervals). In each simulation, the yearly temperature fluctuates following the discretized and truncated normal distribution of the integer type obtained from the Gaussian random variables with mean = *T*_*mean*_ and standard deviation = 0.5 (Fig. S1). We start with the values of the Gaussian probability density function at each integer (from −3 to +3) and then scale them up so that they add up to one. Here discretization and truncation save memory size and simulation time considerably because we only calculate seven integer cases. We expect no qualitative changes with these modifications. The maximum and minimum temperatures are *T*_*mean*_ ± 3. The yearly temperatures have no time correlation (i.e., are independent from each other).

In the original setting, we assume that the ancestral non-periodical cicadas die if they cannot reach maturity by a certain period. An introduced mutant allele is set with this period length. For example, if 10 years are necessary for maturation, the introduced mutant has a 10-year period. Accordingly, when we test the fixation of the 10-year allele, we assume that all non-periodical cicadas die if they do not reach maturation in 10 years. This is rather a severe disadvantage for non-periodical cicadas because they cannot survive over the fixed period length. However, because the climatic conditions during the glacial periods are severe, increasing the juvenile period, juvenile mortality must be increased. Therefore, a periodicity allele should be fixed at the verge of extinction. Nevertheless, this setting might provide a large advantage over non-periodical ancestors. Therefore, we also test the case where this limitation is absent.

### Nymphal growth and accumulated temperatures

Nymphal growth is dependent on accumulated temperatures that are closely correlated with tree growth^7^. Trees grow when the ambient temperature is warmer than their growth limits. The amount of tree growth depends on the difference between the average ambient temperature and the tree growth limit. The yearly growth of trees thus depends on the sum of monthly (or daily) differences accumulated over the year. This sum of the temperatures is often called the effective accumulated (accumulative) temperature. This correlation between tree growth and ambient temperature is suspected to be attributable to water uptake. The water uptake of a tree is expected to cease when the ambient temperature is below the growth limit. Therefore, the rate of tree growth is correlated with the amount of water uptake, which in turn depends on the difference between the average ambient temperature and the tree growth limit.

Here, the amount of cicada growth must also depend on the amount of water taken up by trees because cicadas feed on tree roots. Thus, similar to growth in trees, the growth of cicada nymphs should depend on the accumulated temperatures. Furthermore, the growth limit of a tree should also be variable depending on the microenvironment. If a tree is situated on a south-facing slope, it grows even if the ambient temperature is relatively cold. However, on a north-facing slope, it ceases to grow even if the ambient temperature is relatively warm. Therefore, the growth an individual cicada depends on the microenvironment of the trees it feeds on.

To model climate cooling, we introduce the concept of various temperature variables and parameters.

Here we introduce the concept of lower yearly temperature limit for positive growth for each individual, *T*_*thre,i*_. This means that there are individual variations in both their own growth ability and habitat they location in the yearly temperature threshold of the individual *i*, *T*_*thre,i*_. An individual cicada nymph grows if *T* > *T*_*thre,i*_. We express *T*_*thre-average*_ as the average of *T*_*thre,i*_. We also define *T*_*mean*_, as the average ambient temperature. Then the average relative temperature (ART) is defined as ART = *T*_*mean*_–*T*_*thre-average*_. Note that, when ART = 0, the cicada growth rate is zero (i.e., the cicadas do not grow; Fig. 1a). For each individual, we calculate the sum of the yearly accumulated temperature in the year *t* of individual *i*(*T*_*accume,i*(*t*)_) from the following equation.


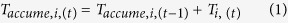


where


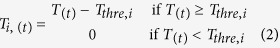


The above variations in the monthly accumulated temperature can be accounted for by the variations in both the temporal environments and the individuals. The individual nymphs mature when the sum of the accumulated temperatures (*T*_*accume,i*(*t*)_) reaches the threshold amount for maturation (*T*_*mature*_). When nymphs reach a cumulative threshold nymphal size, such that *T*_*accume,i*(*t*−1)_ ≥ *T*_*mature*_, they emerge from the ground to become adults. To simulate cold glacial periods, the effective temperatures are set to extremely low levels; as a result, cicada growth to reach emergence size is slow. We observe whether the periodicity allele is fixed in the cicada populations (Fig. 1b). Because mutation is a rare event, we introduce a single period allele per simulation run (i.e., not two or more). Therefore, the words ‘fixed’ and ‘fixation’ are used specifically to mean the acquisition of periodicity (an introduced period allele). Adult cicadas are exposed to the Allee effect, a negative density effect at an extremely low density. We assume that adult populations *N*_*A*_(*t*) cannot reproduce below the critical population size *N*_*c*_.

Thus, we set the growth limit of a cicada based on a yearly temperature threshold (*T*_*thre*_). The cicada nymphs do not grow if the yearly accumulated temperature is below the yearly temperature threshold. There are no additional costs to no growth beyond the yearly mortality and the loss of a year (time). We also introduce the variability of *T*_*thre*_ as follows. The average of the yearly temperature threshold is set to 19, and the variability is *T*_*thre-average*_ ± 2 with equal probabilities, such that *T*_*thre*_ = 17, 18, 19, 20 and 21, with probability = 0.2 for each. Note that the range of ambient temperature variation is set to be ±3 from the average. Because yearly temperature variation represents large-scale climatic change, we assume that the local habitat variation (±2) is smaller than the climatic change. A uniform distribution was chosen here to maintain a high variability in microhabitats and lower the computational loads of the program. We also test discretized and truncated normal distributions with high and low variability (standard deviation = 0.5 and 1.0, respectively) as follows. We start with the values of the Gaussian probability density function at each integer (*T*_*thre*_ = 17, 18, 19, 20, and 21) and then scale them up so that they add up to one (their summation equals one).

### Maturity, emergence and penalty for insufficient growth

Individual nymphs mature when the sum of the accumulated temperature (*T*_*accume,i*(*t*)_) reaches the threshold for maturation (*T*_*mature*_). However, adult emergence differs between periodical and non-periodical cicadas. The ancestral non-periodical cicadas emerge in the year following maturation, such that an adult individual *i*(*t*) emerges if *T*_*accume,i*(*t*−1)_ ≥ *T*_*mature*_. Here, we set *T*_*mature*_ = 10. However, the periodical cicadas necessarily emerge at *Age* = *τ* (period, cycle), irrespective of their maturity (*T*_*accume,i*(*t*−1)_). A female periodical cicada that emerges with insufficient accumulated temperature is penalized in the number of offspring, i.e., *T*_*accume*_(*τ*) < *T*_*mature*_ = 10. We set the basic birth rate *b* = 10 which is the maximum reproductive rate of non-periodical cicadas and matured periodical cicadas. Periodical cicadas with sufficient accumulated temperatures (*T*_*accume,i*_(*τ*) ≥ *T*_*mature*_ = 10) keep the same birth rate with non-periodical cicadas, such that *b*_*p,suff*_ = *b*. The penalty in reproduction incurred by the insufficient accumulated temperature is affected by the amount of shortage in accumulated temperature. Therefore, we assume maturity as the ratio of the accumulated temperature (*T*_*accume,p,i*_) to the temperature necessary for maturation (*T*_*mature*_), such that


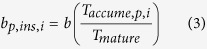


### Yearly nymphal mortality

All nymphs suffer from nymphal mortality every year. We set the yearly mortality with density effects such that


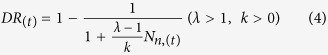


where *DR*_(*t*)_ is the mortality rate of year *t*, and *N*_*n*_ is the population size of the nymphs following the Beverton-Holt model^26^. We set the two model parameters (λ = 1.005 and *k* = 250: red line) for most simulation runs (Fig. S2). We also test the different parameter conditions (λ and *k*) because these parameters may be critical for the outcomes of the simulation (see supplementary text).

## Results

**Figure 2:**
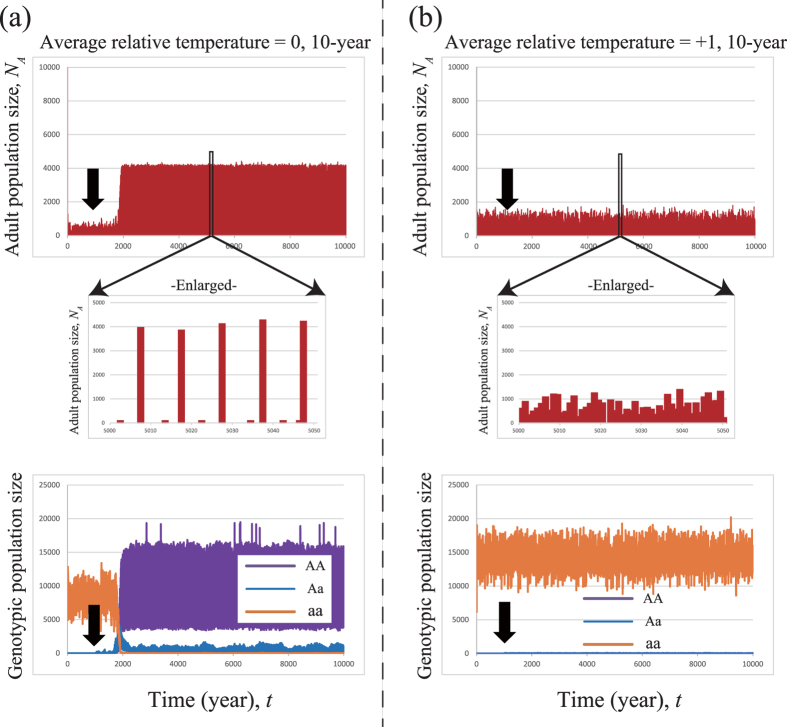
Temporal dynamics of cicada populations with 10-year periodicity introduced under cool and warm ambient environments. (**a**) Cool environments (ART = 0) and (**b**) warm environments (ART = +1). (top) Annual adult population sizes. (middle) Enlargement of top for *t* = 5000~5050. (bottom) Annual total population sizes for each genotype (*AA*: purple, *Aa*: blue, *aa*: orange). The black arrow points to the first step (*t* = 1,000) when the mutation is introduced. The extinction threshold of the Allee effect is set as *N*_*c*_ = 100. The parameters for annual mortality are *k* = 250 and *λ* = 1.005. The penalty of reproduction for immature periodical adults is set to be dependent on the shortage of accumulated temperature.

**Figure 3:**
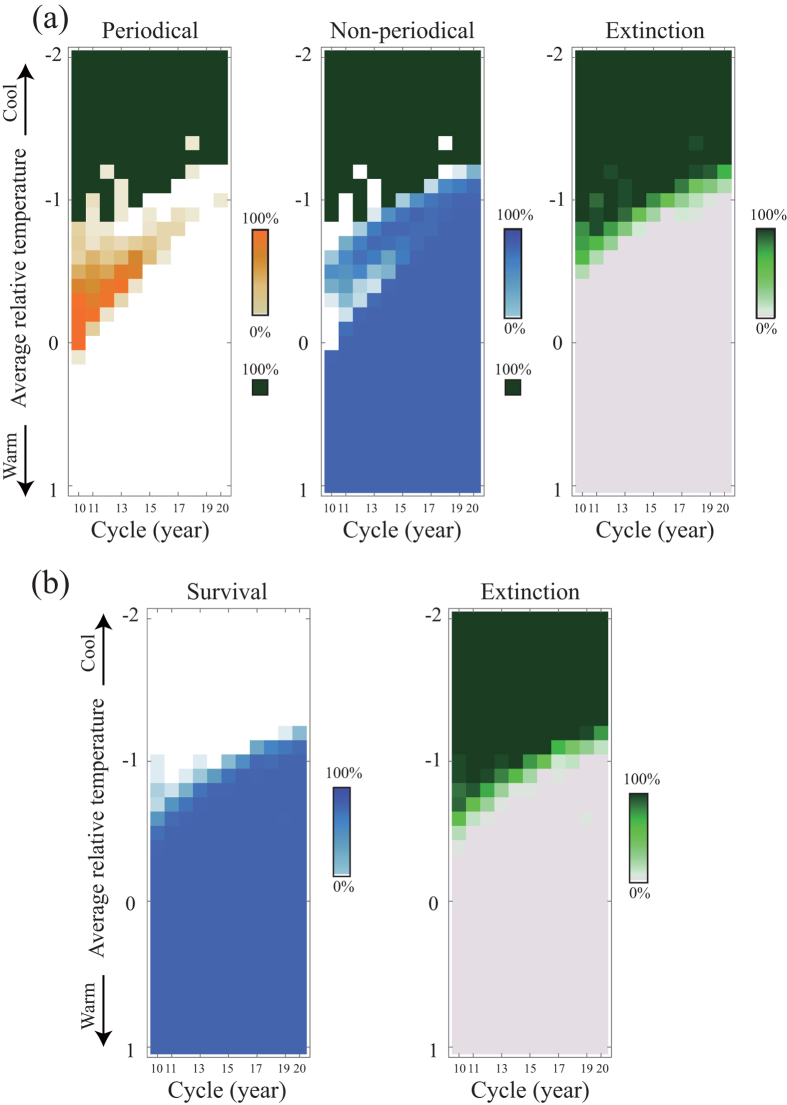
Phase diagrams of 10- to 20-year periodicity introductions versus average relative temperature (ART). (**a**) Mutation experiment (**left**: fixation; **middle**: no fixation; **right**: extinction). (**b**) Control (no mutation) (**left**: survival; **right**: extinction). Green areas: extinction; orange: fixation of periodicity; blue: no fixation (darker colours indicate higher probabilities). The extinction threshold of the Allee effect is set as *N*_*c*_ = 100. The parameters for annual mortality are *k* = 250 and *λ* = 1.005. The penalty of reproduction for immature periodical adults is set to be dependent on the shortage of accumulated temperature. The results are based on 50 simulation runs for each condition.

The simulation results show that fixation of the periodicity allele is frequently observed some time after the introduction of the mutation (Fig. 2). Here we show the population dynamics of a single simulation run. In a cooler climate (ART = 0), temporal dynamics indicate that when the 10-year periodicity allele mutation is introduced at *t* = 1,000 under the adult extinction threshold *N*_*c*_ = 100, the fixation of periodicity occurs at *t* = 2,000 (Fig. 2a). However, in a warm climate (ART = +1), no fixation is observed (Fig. 2b). When fixation occurs, the average adult population size increases approximately threefold, from less than 500 adults emerging annually to over 4,000 adults in a mass emergence (Fig. 2a). We also assessed the emergence years and broods. When a periodicity allele is fixed, only one brood is fixed (Fig. 2a (middle)). In contrast, when periodicity is not fixed, annual emergence is seen with variability in adult numbers, but no mass emergence occurs (Fig. 2b (middle)). We also investigated the genotypic population sizes, including the juveniles (Fig. 2a (bottom) and 2b (bottom)). In the cooler climate, the total population size increases from approximately 10,000 to approximately 15,000 individuals (Fig. 2a (bottom)). Here, almost all individuals are homozygous for the periodical alleles of one brood at the locus determining the timing of emergence. In contrast, in the warmer climate, no periodicity is fixed, not even with the shortest periodicity of 10 years (Fig. 2b). In stochastic models, without an example of a simulation run, it is difficult for readers to imagine how the temporal dynamics looks like. We actually run the simulations several times. The qualitative results are summarized in Fig. 3 based on 50 runs. For example, when the periodicity is fixed (Fig. 2a), all fifty runs show fixation (the left column in Fig. 3a), even though the timing of fixation is different among simulations (Table 1).

We examined the simulation dynamics by varying the introduced periodicity from 10 to 20 years and the ART from +1 to −2 at step = 0.1 (Fig. 3). There is a border of extinction such that if the climate is cooler than the threshold, the population usually goes extinct. However, some cicadas survive under such conditions in a small number of runs. If the climate is warmer than this threshold, cicadas survive in almost all simulation runs. Periodicity is fixed at and near the boundary of extinction. A wide band of fixed periodicity is found slightly below the boundary of extinction, where the shorter (longer) cycles are fixed at warmer (cooler) cumulative temperatures. A small band of 10–12 years is also observed at the boundary of extinction (Fig. 3a). As a control, we also ran the simulation without introducing mutations (Fig. 3b). The surviving regions of both experiments and controls largely agree (the extinction region is almost identical in Fig. 3a (right) and 3b (right)). However, under short life cycles, the conditions under which cicadas survive are slightly broader than those of the controls. We also note that some extinction is observed slightly below the complete extinction borders (ART = −0.5 to 0.0). The survival region above this area (ART = −1.0 to −0.5) might be in the extinction zone (green area in Fig. 3), but survival in this region appears to be possible due to a combination of cycles, growth rates and initial conditions.

We also tested various extinction conditions. In the current models, all non-periodical nymphs that cannot mature within the introduced periods are assumed to die. First, we removed the period limitation of longevity for non-periodical cicadas, allowing non-periodical nymphs to survive until 50 years, i.e., the maximum longevity setting. Surprisingly, this removal of the limitation of juvenile longevity greatly relaxed the fixation condition for periodical cicadas (Fig. S3 vs. Fig. 3). When the extinction threshold *N*_*c*_ was lowered from *N*_*c*_ = 300 to *N*_*c*_ = 100 and *N*_*c*_ = 0, the region of periodicity fixation was drastically enlarged (Fig. S4). However, the thresholds of extinction at *N*_*c*_ = 100 and *N*_*c*_ = 300 are almost identical, and the trend of fixation of longer cycles at lower temperatures was similar. We also inspected the case when the Allee effects are variable depending on the adult population size *N*_*A*_. We found a slight decrease in the area of fixation but no qualitative differences (Fig. S5 vs. Fig. 3). We also tested various parameter *k* values of annual mortality rate (Fig. S6). Mortality may be affected by yearly temperatures, such that lower temperatures increase nymphal mortality. Freezing temperatures will not only reduce the amount of root water flow available to the nymphs but also promote nymph desiccation, resulting in high nymph mortality. We assume that a cool environment has a low *k* (=200). In contrast, a warm environment has a high *k* (=300). In this case, extinction occurred under conditions of much higher ART, resulting in the extinction of longer cycles (16–20 years). We also assume that a cool environment has a high *λ* parameter value (=1.007). In contrast, a warm environment has a low *λ* (=1.003). Here, we found almost no qualitative changes in the outcomes (Fig. S7).

In our simulation, we assumed that the periodicity allele is dominant, whereas the temperature-dependent allele is recessive (Figs 2 and 3). We also tested the fixation of the periodicity allele when the temperature-dependent allele is dominant (i.e., the periodicity allele is recessive) under a wide range of mutation rates (0.001 to 0.01). Under this condition, the fixation of periodicity is observed when the mutation rate is sufficiently large (Figs. S8, S9). We also found that shorter periods are associated with the fixation of periodicity even at even low mutation rates. We also tested the fixation of recessive periodicity allele when the period limitation of longevity for non-periodical cicadas is removed (Fig S3). The periodicity fixation is observed in a wider range of average relative temperature (e.g., Fig. S10 for 10-year cycle), compared to those with period limitation (Fig. S8a). Thus, the dominant/recessive nature of the periodicity allele quantitatively affects the threshold mutation rates for periodicity evolution, but it does not affect the evolution of periodicity itself (Figs S8, S9, S10). The evolution of periodicity should occur over a long run, irrespective of allele dominance, as long as extinction does not occur. Here, we present results based on the condition that an individual cannot have multiple independent mutations. The results are approximately the same with multiple independent mutations (Fig. S11). We also tested discretized and truncated normal distributions with high and low variability (standard deviation = 0.5 and 1.0, respectively) that more closely resemble the natural condition of habitat variation. We found no qualitative changes (Fig. S12).

## Discussion

Our computer simulation demonstrates that the evolution of periodicity^20^ is possible under highly restricted conditions. If our model accurately captures the evolution of periodicity in *Magicicada,* then cooling during glacial periods^27^ must have been a critical factor in *Magicicada* evolution and the conditions in glacial refugia must have been similar to those in our model. Surprisingly, longer life cycles are not fixed at the boundary of extinction, probably because population sizes under such conditions are too small to permit the fixation of periodical cycles (Fig. 3a). In shorter cycles, fixation may be achieved at the verge of extinction because the cicadas with a shorter cycle reproduce more frequently (Fig. 3a). When the total time steps are increased from 10,000 to 30,000, the results are almost identical, indicating that 10,000 steps are sufficient to reach a steady state in the current simulation (Fig. S13).

In our model, when switching to periodicity takes place, the periodical cicadas converge to a single brood (Fig. 2a (middle)). In the initial stages of evolution, there may be a few broods (different emergence-year cycles) of periodical mutants; however, only one brood survives and all other founder broods appear to be eliminated by Allee effects and yearly nymphal mortality. All smaller founder broods suffer strong Allee effects, and only the largest of the broods appear to avoid Allee effects and persist. Without Allee effects (*N*_*c*_ = 0), a few small broods persist alongside one large brood for a while, but they are eventually eliminated by the constant annual nymphal mortality (Fig. S9c vs. Fig. 2a). In contrast, even under weak Allee effects (*N*_*c*_ ≤ 50), small founder broods are immediately eliminated. The current accumulated data on the distribution of periodical cicadas suggest that broods are largely non-overlapping^28^,^29^. Such habitat exclusion, i.e., one location-one brood, may be the result of mechanisms similar to those in our model. We also observe a drastic increase in adult emergence and maximal nymphal population sizes (Fig. 2a (bottom) and 2b (bottom)). If our model dynamic is realistic, then the extraordinary mass emergence of periodical cicadas may be partly attributed to the processes involved in the evolution and fixation of periodicity.

Under stronger Allee effects (from *N*_*c*_ = 100 to *N*_*c*_ = 300), the extinction threshold at which periodicity is acquired is lowered (Fig. S4). This pattern may arise because the rare periodicity mutants in small populations are eliminated by the Allee effects, particularly during the early stages, which sharply reduces the fixation probability of the periodicity allele. In fact, if the Allee effect is weak or non-existent (*N*_*c*_ = 0), periodicity spreads under a wide range of conditions. Similarly, as a periodical life cycle becomes longer, the probability of periodicity acquisition gradually lowers due to several reasons: First, cicadas with longer life cycles are exposed to cumulative annual mortality more times than are those with shorter life cycles, and the effects of cumulative mortality are reset less often by opportunities for reproduction. Second, the adult emergence tends to spread out in longer cycles because of the unevenness of juvenile growth is increased among individuals. This results in a lower probability of fixation in longer cycles. Above all, there are fewer opportunities for periodicity allele fixation in longer cycles because there is a lower total number of life cycles (generations) per given period. For example, 10-year cicadas reproduce twice in a 20-year period, whereas 20-year cicadas reproduce only once during this time. Cumulative mortality and limited mating opportunities may be one of the primary reasons why 19-year cicadas are not observed in nature.

This simulation model is not a dynamic model with gradual geological cooling; rather, it is a stochastic dynamic model with yearly variation under a range of cold climates. We did not use a dynamic model because the yearly stochastic variation in climates is several magnitudes larger than the geological cooling. By testing a wide range of cold climates, we can evaluate the effects of geological cooling without dynamic geological models. Our model shows that there are two important components of randomness that contribute to the acquisition of periodicity: (1) yearly stochasticity in climate and (2) variations in microhabitats and individual growth (see Fig. S14). The acquisition of periodicity at and near the boundary of extinction suggests that geological cooling should have contributed to the fixation of periodicity in ancestral cicadas with cumulative temperatures.

Our simulation demonstrates the feasibility of the first step of Yoshimura’s (1997) hypothesis for the evolution of periodicity during glacial periods. Our results indicate that various year cycles (10–20 year) could have evolved depending on slight differences in accumulated temperatures. The fixation of periodicity is also exhibited at and near the boundary of extinction, as in the case of selection for prime-numbered cycles^21^. Interestingly, the fixation of periodicity is more likely if the Allee effect is non-existent or weak (i.e., *N*_*c*_ = 0 or 100), whereas the selection of prime-numbered cycles is promoted by a stronger Allee effect^22^. Therefore, an intermediate Allee effect may be necessary for the evolution of prime-numbered cycles in periodical cicadas. The current results also agree with the approximate distribution of the 17-year cicadas, which are north of the 13-year cicadas.

Previous models have not considered the life history characteristics of the ancestral periodical cicadas^4^,^19^,^20^. Although all known non-periodical cicadas experience temperature-dependent growth rates and size-dependent maturation, with adult emergence triggered by the threshold of accumulated temperature (*T*_*mature*_ in Fig. 1a), the periodical cicadas are the only known cicadas to exhibit time-dependent maturation. The current results suggest that the evolution of periodicity is plausible, but only under restricted conditions near or at the extinction boundary.

Many periodical organisms are known in the world, e.g., cicadas^16^,^17^,^18^ and plants^30^,^31^,^32^. *Magicicada* is associated with prime-numbered life cycles. However, well-known bamboo plants are estimated to have 15-, 30-, 60-, and 120-year life cycles, and possibly more, almost all of which are non-prime cycles^30^,^32^. Recently, a subtropical plant, *Strobilanthes flexicaulis*, was confirmed to have a 6-year life cycle^31^. Fijian and Indian cicadas exhibiting mass emergence are also suspected to be periodical under shorter life cycles^16^,^17^,^18^. If these cicadas are periodical, their evolutionary mechanism for periodicity should be different from that of the *Magicicada* group. The 17- and 13-year life cycles of the *Magicicada* group are unique among all periodical organisms because of their extremely long, prime-numbered cycles.

## Additional Information

**How to cite this article**: Ito, H. *et al.* Evolution of periodicity in periodical cicadas. *Sci. Rep.* **5**, 14094; doi: 10.1038/srep14094 (2015).

## Supplementary Material

Supplementary Information

## Figures and Tables

**Table 1:** List of Parameters.

Term	Unit	Explanation
*t*	Year	Time step (year)
*T*	°C	Yearly ambient temperature (random)
*T*_*mean*_	°C	Average of yearly ambient temperature
*τ*	Year	Cycle (period) length
*T*_*thre*_	°C	Lower yearly temperature limit for positive growth
*T*_*thre-average*_	°C	Average of yearly temperature threshold is set to 19 and the variability is *T*_*thre-average*_ ± 2 with equal probabilities
*T*_*accume*_	°C	Sum of yearly accumulated temperature
*T*_*mature*_	°C	Amount of the accumulated temperature required for maturation (adult emergence)
ART	Δ °C	Average relative temperature = (*T*_*mean*_ − *T*_*thre-average*_)
*N*_*c*_	No. individuals	Extinction threshold for adult populations set by the Allee effect
*N*_*n*_	No. nymphs	Population size of nymphal cicadas
*N*_*A*_	No. Adults	Population size of adult cicadas
*b*	No. eggs/Adult	Birth rate is set as *b* = 10
*b*_*p,suff*_	No. eggs/Adult	Birth rate of a periodical adult female with sufficient accumulated temperature (*T*_*accume*_(*τ*) ≥ T_mature_ = 10)
*b*_*p,ins*_	No. eggs/Adult	Birth rate of a periodical adult female with insufficient accumulated temperature (*T*_*accume*_(*τ*) < *T*_*mature*_ = 10)
*DR*(*t*)	Probability	Mortality rate at time *t* (probability)
*k*		Parameter of yearly nymphal mortality (*DR*(*t*))
*λ*		Parameter of yearly nymphal mortality (*DR*(*t*))
