# Supplementary material for: Evolution of periodicity in periodical cicadas
Source: Sci Rep. 2015 Sep 14;5:14094. doi: 10.1038/srep14094 (PMC4568538; doi:10.1038/srep14094)
Supplement: Supplementary Information [file srep14094-s1.pdf]

Evolution of periodicity in periodical cicadas
Supplementary Information

Authors: Hiromu Ito[1], Satoshi Kakishima[1], Takashi Uehara[1,2], Satoru Morita[3], Takuya Koyama[4], Teiji Sota[4], John R. Cooley[5], Jin Yoshimura[1,3,6,7,*]

[1]Graduate School of Science and Technology, Shizuoka University, Hamamatsu, 432-8561, Japan
[2]Nagoya College, Toyoake, Aichi Pref., 470-1193, Japan
[3]Department of Mathematical and Systems Engineering, Shizuoka University, Hamamatsu, 432-8561, Japan
[4]Department of Zoology, Graduate School of Science, Kyoto University, Sakyo, Kyoto 606-8502, Japan.
[5]Department of Ecology and Evolutionary Biology, University of Connecticut, Storrs, CT 06268-3043, USA
[6]Department of Environmental and Forest Biology, State University of New York College of Environmental Science and Forestry, Syracuse, NY 13210 USA
[7]Marine Biosystems Research Center, Chiba University, Uchiura, Kamogawa, Chiba 299-5502, Japan

Supplementary text
Description of the Individual Based Model for the evolution of periodicity in periodical cicadas

Outlines:

- **Setup of individuals**
- **Setup of yearly nymphal mortality**
- **Simulation flow**
- **Supplementary figures**

● Setup of individuals

Each individual is associated with the following information (trait values):

1. Periodicity alleles

The locus for the determinant of maturation has two different alleles: a temperature (size)-dependent allele (*a*) and a time-dependent (periodicity) allele (*A*). We assume that the periodicity allele (*A*) is dominant and the non-periodicity allele (*a*) is recessive. Therefore, individuals with *AA* and *Aa* genotypes emerge periodically, whereas those with *aa* genotypes emerge when their larval size exceeds a certain threshold (mature size). The genotypes of the ancestral cicadas are *aa*, and those of *AA* and *Aa* are periodical cicadas. We also evaluate the evolution of periodicity assuming that the periodicity allele is recessive and the non-periodicity allele is dominant. Simulation runs begin with all individuals having only temperature-dependent alleles; then, when the ancestral cicada population reaches a low stable density (step t=1,000), bidirectional mutation at the emergence locus is introduced at the rate of 0.001 per locus. We also test the case where the temperature-dependent allele is dominant under varying mutation rates (0.001 to 0.01).

When we introduce periodicity alleles, τ-year period is introduced for τ=10-20. Within each simulation, only a single τ-year periodicity is introduced. We tested 10- to 20-year periods separately. The remaining year before emergence is also recorded for periodical cicadas. In the initial conditions, all individuals are set to have non-periodicity alleles. We introduced mutation at t=1000 (year) when the steady-state densities are reached. For each allele, bidirectional mutation is introduced at the birth of an individual at the rate of 0.001 from time step t=1,000 to the end of simulation steps ($a \leftrightarrow A$). We also run the simulation assuming one mutation per individual, such that no individual has a simultaneous double mutation (Fig. S11).

2. Age and virtual longevity

An egg has an age of zero years when it is deposited. Here, we assume that all non-periodical nymphs die if they do not reach maturity before the introduced period length (τ year). We also evaluate the effect of relaxing this condition by assuming that each individual nymph has the maximum virtual longevity of 50 years (Age_{max}=50 years). In warm climates, all cicadas should have emerged as adults or died before this age limit. However, in very cold climates, many nymphs never reach the adult stage and die with this 50-year age limit (see Fig. S3). The age (*Age*) of non-periodical cicadas ranges from 0 to 50, whereas that of periodical cicadas ranges from 0 to τ (τ, periodicity of emergence; $10 \le \tau \le 20$) because all periodical cicadas that reach maturity emerge as adults.

● Setup of yearly nymphal mortality

Yearly nymphal mortality is set following the Beverton-Holt model[32]. In this model, there are two

parameters, λ and *k*. The original settings are used for these two parameters (λ=1.005 and *k*=250: red line) for most simulation runs (Fig. S2). Mortality may be affected by yearly temperatures, with lower temperatures increasing nymphal mortality. Freezing temperatures will not only reduce the root water flow available to the nymphs but also promote nymph desiccation, resulting in high nymph mortality. Therefore, we also vary these parameters to determine their effects. We first test various *k* values (*k*=200, 250, 300; supplementary Fig. S6). We assume that a cool environment has a low *k* parameter value (=200). In contrast, a warm environment has a high *k* (=300). We also vary λ (λ=1.003, 1.005, 1.007) (Fig. S6, S7).

- **Simulation flow**

The computer program is the individual-based model (IBM or multi-agent model), where all individuals are tracked during each time step. We only consider a female population and the corresponding male population is assumed to be identical, except for the penalty of birth rates, which is only associated with females. The simulation procedures are as follows:

1) Initially, we set the population size at 10,000 individuals, i.e., $N_A(t=0)=10{,}000$. All individuals are non-periodical cicadas with genotype *aa*.

2) Adult reproduction and nymphal growth are as follows:

(1) Adult cicadas mate and reproduce with a given birth rate. The birth rate is set as $b=10$ or as otherwise specified. All adults die immediately after reproduction.

(2) All eggs hatch immediately to become nymphs of age ‘zero’ years.

(3) The 0-year nymphs suffer no annual mortality.

(4) The nymphs of each age (L_{Age} for $Age=1, \ldots, 49$) suffer from annual mortality *DR*.

(5) The 50-year nymphs that do not reach maturity die before emerging as adults.

(6) The mature nymphs emerge to become adults.

(7) If the total number of emerging adults is less than the critical population size N_c, no reproduction takes place, resulting in extinction. However, if the total number exceeds N_c, return to step (1) and repeat the nymphal growth process for up to 999 time steps.

3) Upon the 1000th step, we introduce a bidirectional mutation between the periodicity and non-periodicity alleles (“*A*”↔”*a*”) in the offspring population at a mutation rate of 0.001 when reproduction takes place.

4) Emergence takes place as follows. Non-periodical nymphs emerge when they mature ($T_{accume,i}(t) \geq T_{mature}=10$). Periodical nymphs become adults when they reach the given period (τ year). A female periodical cicada emerging with insufficient accumulated temperature ($T_{accume}(\tau) < T_{mature}=10$) is given a discounted birth rate (i.e., $b_{p,ins,i}$).

5) Mating takes place at random among all emerged individuals. The genotypes of the offspring follow Mendelian inheritance.

6) Repeat steps 2) to 5) for an additional 9,000 time steps until t=10,000 or otherwise specified.

- **Supplementary figures and legends**

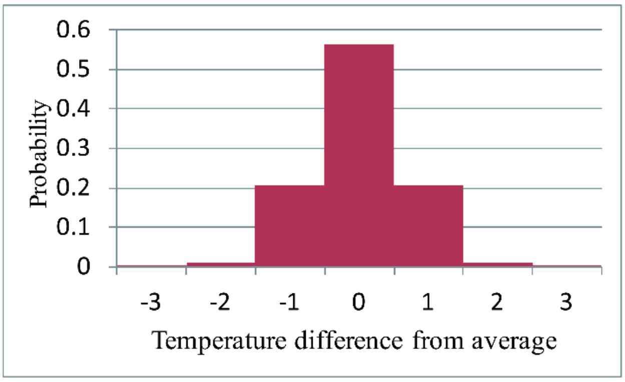

Fig. S1. Probability of yearly ambient temperature deviations from the average temperature. Bar graph expressing the event probability of a yearly temperature deviating from the average temperature.

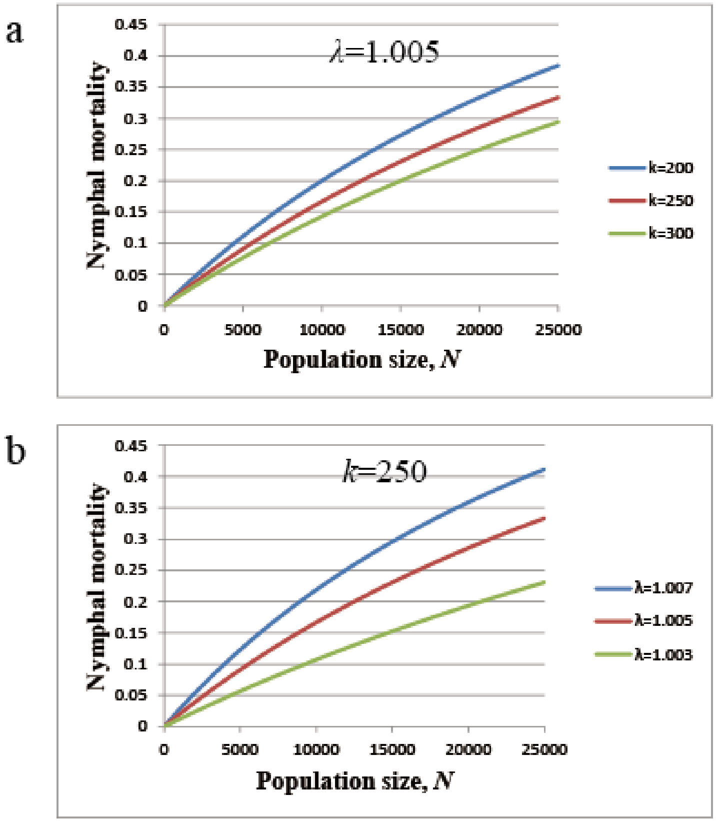

Fig. S2. Density-dependent nymphal mortality following the Beverton-Holt (1957) model with two parameters, λ and *k*. (**a**) A cool environment has a low *k* parameter value (=200). In contrast, a warm environment has a high *k* (=300). (**b**) A cool environment has a high λ(=1.007). In contrast, a warm environment has a low λ (=1.003).

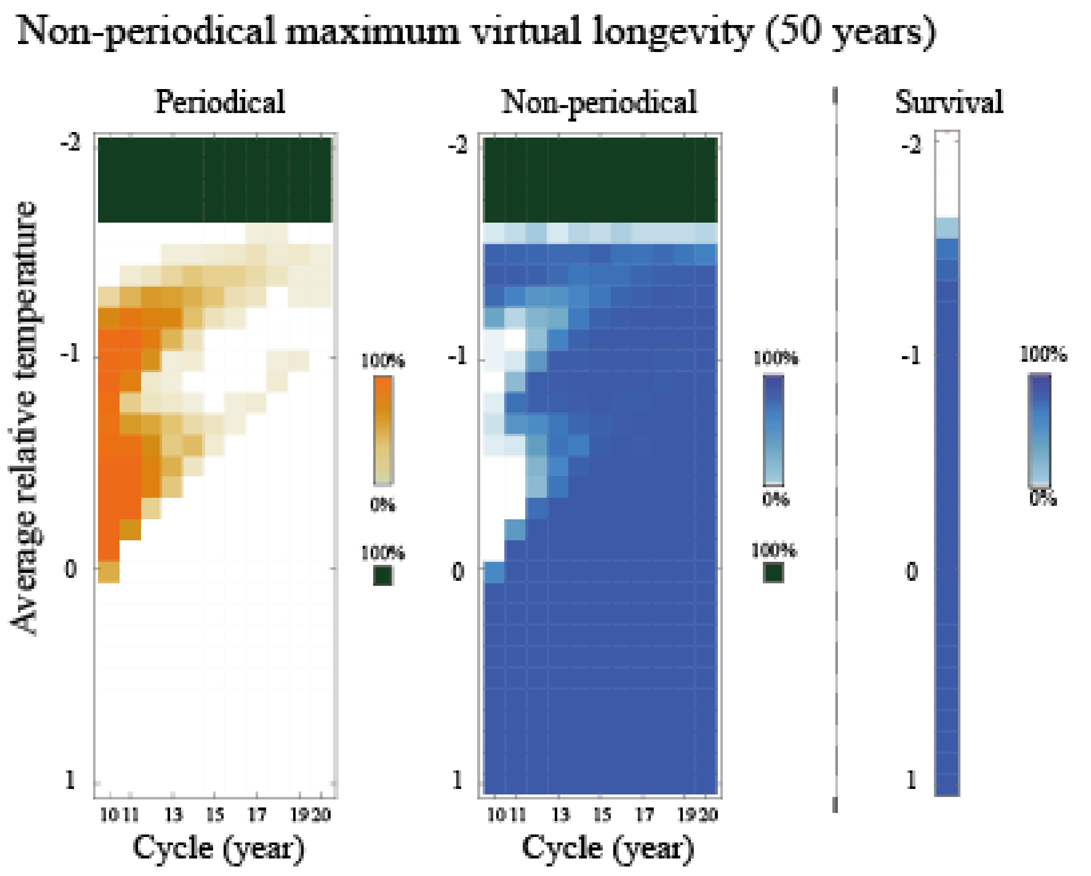

Fig. S3. Phase diagrams of 10- to 20-year periodicity introductions versus average relative temperature (ART) when the longevity of non-periodical cicada nymphs is 50 years. (**left, middle**) Mutation experiment (**left**: fixation; **middle**: no fixation). (**right**) Control (no mutation introduced, survival). Green areas: extinction; orange: fixation of periodicity; blue: no fixation (darker colours indicate higher probabilities). The results are based on 50 simulation runs for each condition. The Allee effect is set as the critical population size N_c, below which the population is set to extinction, such that N_c=100. The annual mortality parameter is set as k=250, λ=1.005. The penalty of reproduction due to immature periodical cicadas is set to be dependent on the shortage of accumulated temperature.

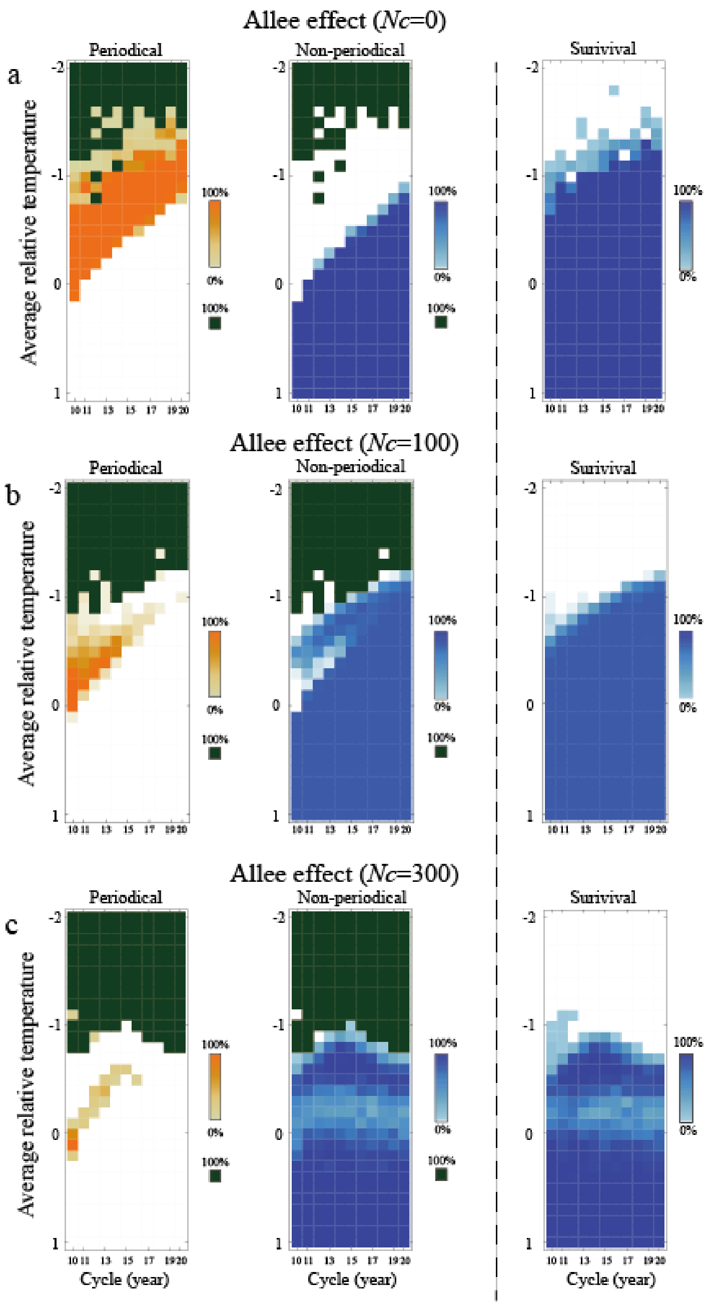

Fig. S4. Phase diagrams of 10- to 20-year periodicity introductions versus average relative temperature (ART) with three different levels of extinction threshold N_c for the Allee effects. (**a**) N_c=0, (**b**) N_c=100, (**c**) N_c=300. (**left, middle**) Mutation experiment (**left**: fixation; **middle**: no fixation). (**right**) Control (no mutation, survival). Green areas: extinction; orange: fixation of periodicity; blue: no fixation (darker colours indicate higher probabilities). The parameters for annual mortality are k=250 and λ=1.005. The penalty of reproduction due to immature periodical cicadas is set to be dependent on the shortage of accumulated temperature. The results are based on 50 simulation runs for each condition.

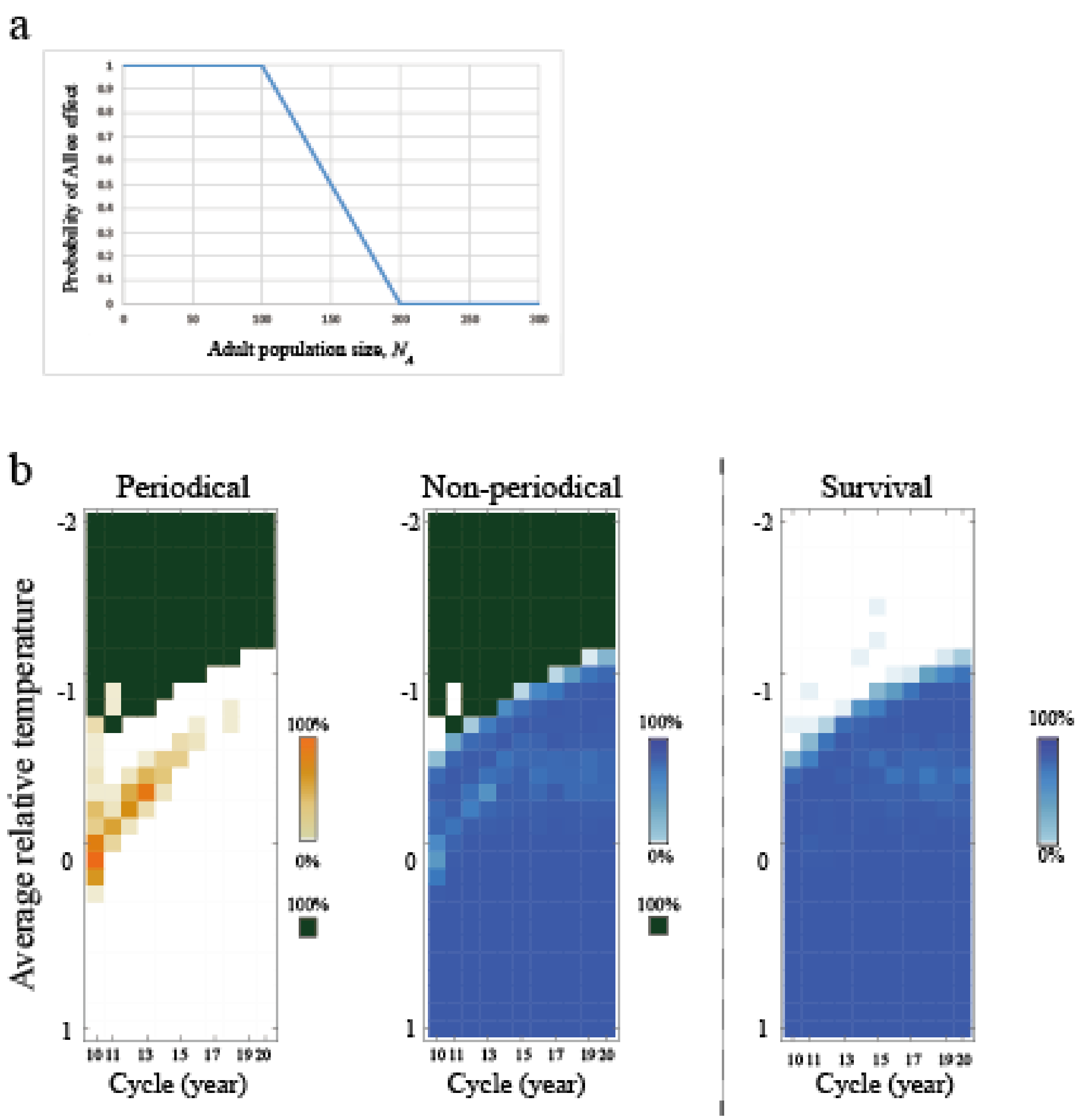

Fig. S5. Phase diagrams of 10- to 20-year periodicity introductions versus average relative temperature (ART) with varying degrees of extinction thresholds N_c for the Allee effects. (**a**) Probability of the Allee effect (N_c), i.e., $p(N_c=N_A)$. If $N_A<100$, $p=1$; if $100\leq N_A\leq 200$, $p=- N_A/100+2$; and if $N_A>200$, $p=0$. (**b**) (**left, middle**) Mutation experiment (**left**: fixation; **middle**: no fixation). (**right**) Control (no mutation, survival). Green areas: extinction; orange: fixation of periodicity; blue: no fixation (darker colours indicate higher probabilities). The parameters for annual mortality are $k=250$ and $\lambda=1.005$. The penalty of reproduction due to immature periodical cicadas is set to be dependent on the shortage of accumulated temperature. The results are based on 50 simulation runs for each condition.

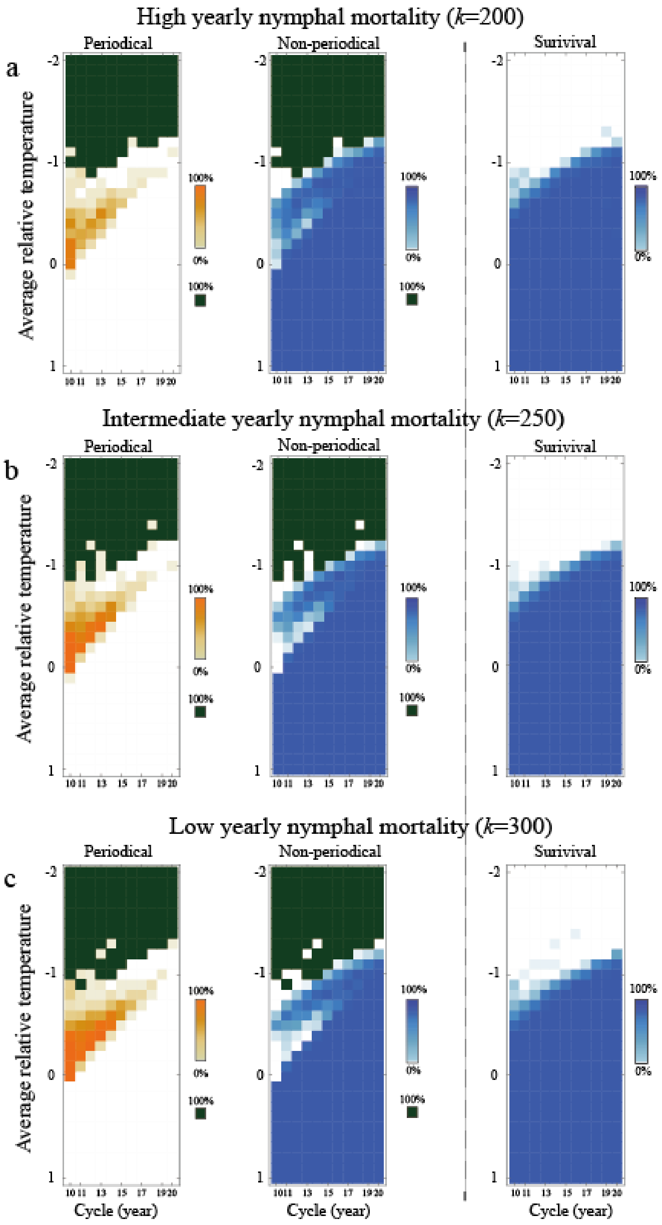

Fig. S6. Phase diagrams of 10- to 20-year periodicity introductions versus average relative temperature (ART) with three different yearly nymph mortality rates. Parameter k is varied, while parameter λ is held constant (λ=1.005). (**a**) k=200, (**b**) k=250, (**c**) k=300. (**left, middle**) Mutation experiment (**left**: fixation; **middle**: no fixation). (**right**) Control (no mutation, survival). Green areas: extinction; orange: fixation of periodicity; blue: no fixation (darker colours indicate higher probabilities). The extinction threshold of the Allee effect is set as N_c=100. The penalty of reproduction due to immature periodical cicadas is set to be dependent on the shortage of accumulated temperature. The results are based on 50 simulation runs for each condition.

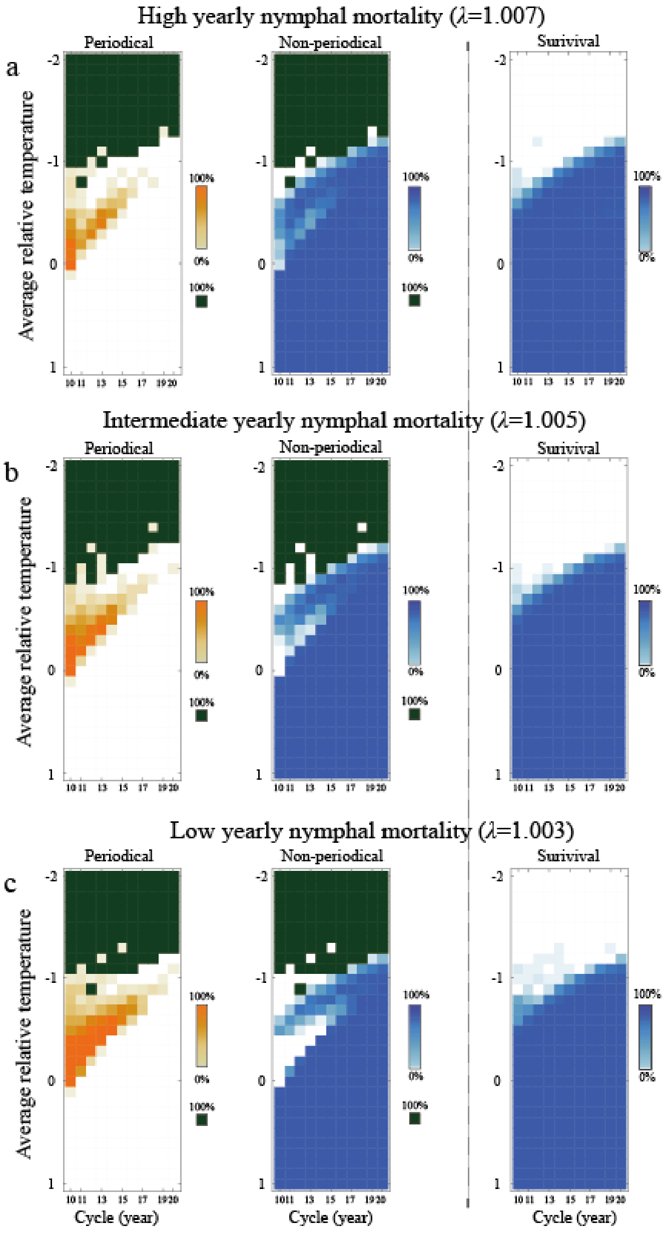

Fig. S7. Phase diagrams of 10- to 20-year periodicity introductions versus average relative temperature (ART) with three different yearly nymph mortality rates. Parameter λ is varied, while parameter k is held constant (k =250). (**a**)λ=1.007, (**b**) λ=1.005, and (**c**) λ=1.003. (**left, middle**) Mutation experiment (**left**: fixation; **middle**: no fixation). (**right**) Control (no mutation, survival). Green areas: extinction; orange: fixation of periodicity; blue: no fixation (darker colours indicate higher probabilities). The extinction threshold of the Allee effect is set as N_c=100. The penalty of reproduction due to immature periodical cicadas is set to be dependent on the shortage of accumulated temperature. The results are based on 50 simulation runs for each condition.

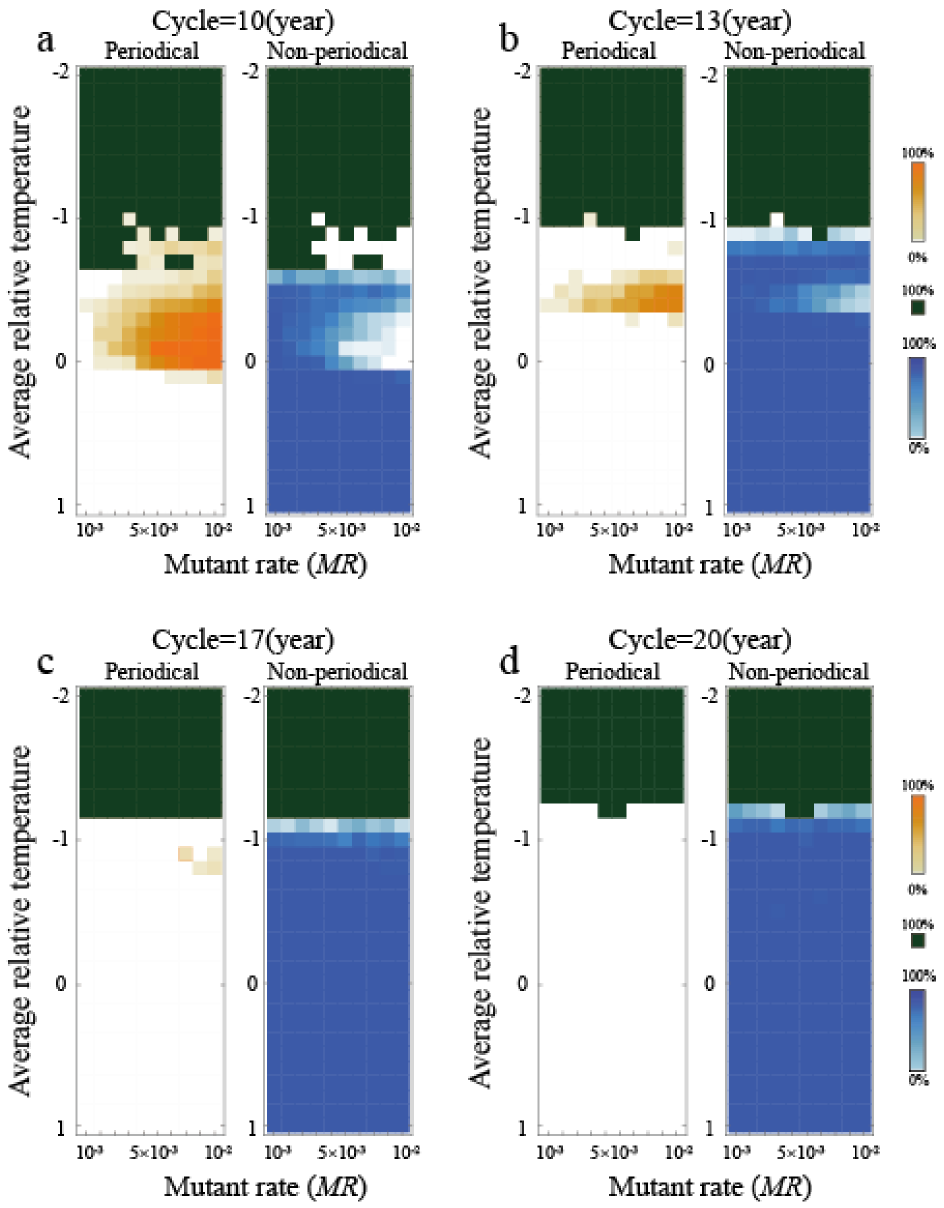

Fig. S8. Phase diagrams of periodicity introduction with varying levels of mutation rates versus average relative temperature (ART) with four different periods when the non-periodical allele is dominant. (**a**) 10-year period, (**b**) 13-year period, (**c**) 17-year period, (**d**) 20-year period. Mutation rates range from 10^{-3} to 10^{-2} (interval=10^{-3}). **Left**: fixation; **right**: no fixation. Green areas: extinction; orange: fixation of periodicity; blue: no fixation (darker colours indicate higher probabilities). The extinction threshold of the Allee effect is set as N_c=100. The parameters for annual mortality are k=250 and λ=1.005. The penalty of reproduction for immature periodical adults is set to be dependent on the shortage of accumulated temperature. The results are based on 50 simulation runs for each condition.

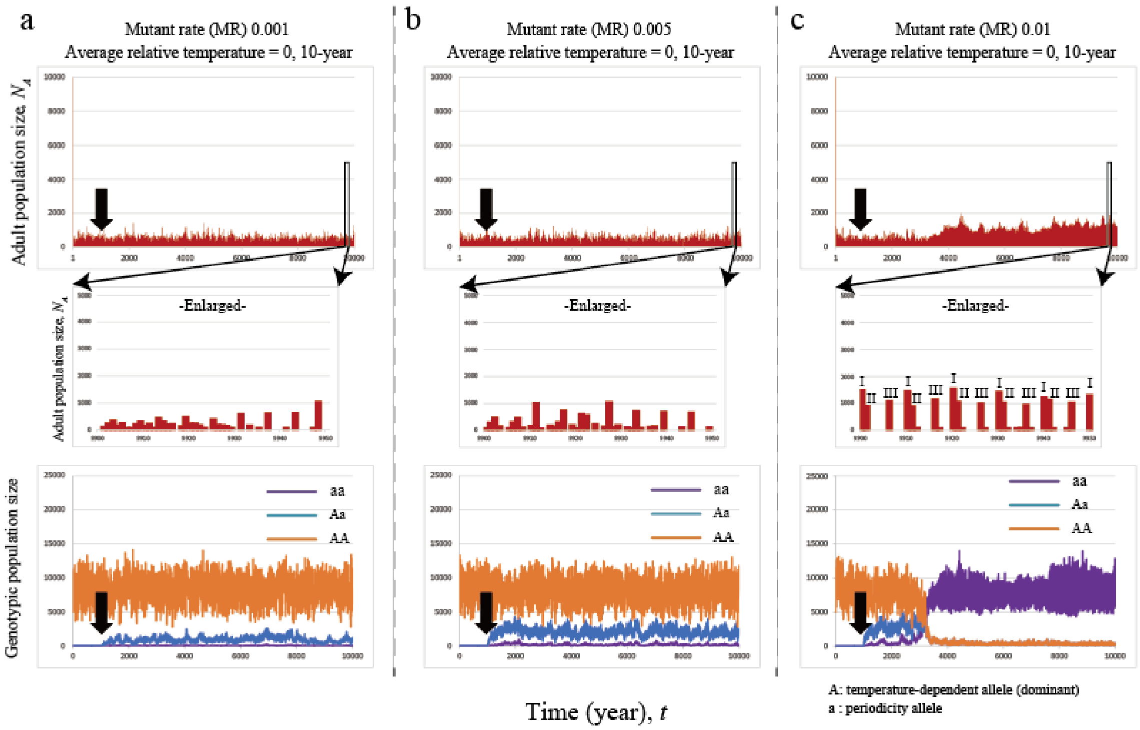

Fig. S9. Temporal dynamics of cicada populations with 10-year periodicity introduced and the non-periodicity allele set as dominant. (**a**) Mutation rate=0.001, (**b**) mutation rate=0.005, and (**c**) mutation rate=0.01. (**top**) Annual adult population sizes. (**middle**) Enlargement of **top** for 9900~9950, including three different broods (Broods I, II, and III). (**bottom**) Annual total population sizes for each genotype (*AA*: purple, *Aa*: blue, *aa*: orange). The black arrow points to the first step (t=1,000) when the mutation is introduced. The extinction threshold of the Allee effect is set as N_c=100. The parameters for annual mortality are k=250 and λ=1.005. The penalty of reproduction for immature periodical adults is set to be dependent on the shortage of accumulated temperature.

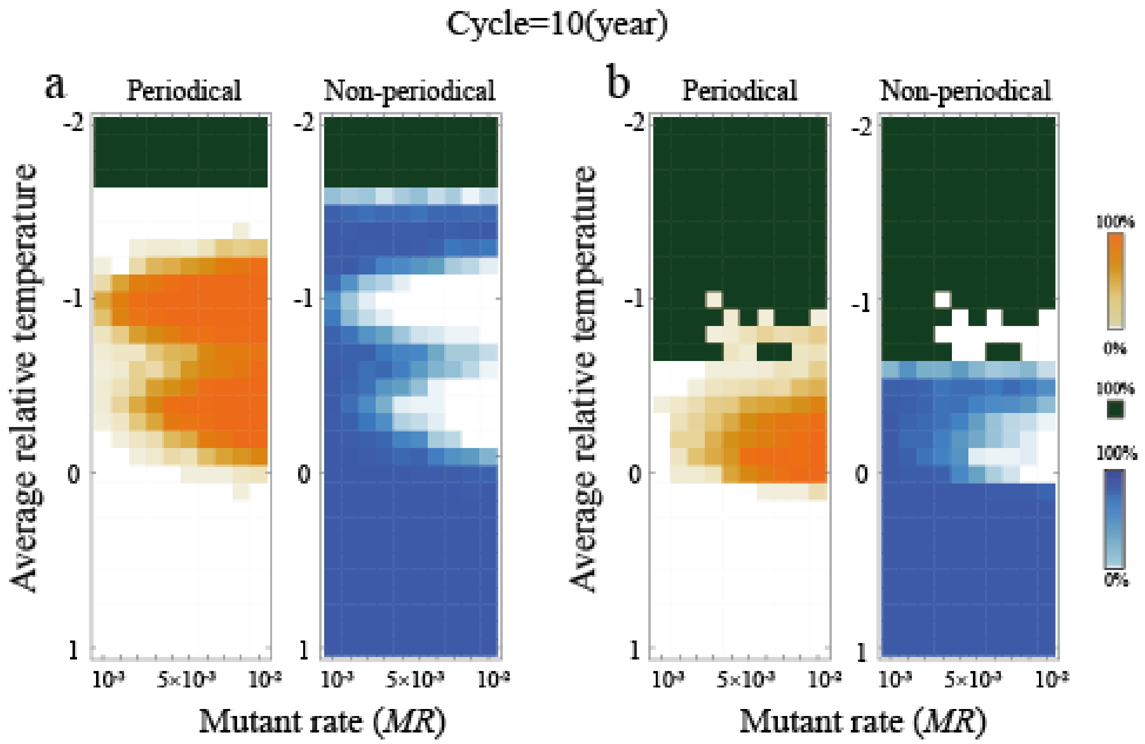

Fig. S10. Phase diagrams of periodicity introduction with varying levels of mutation rates versus average relative temperature (ART) with 10-year period when the non-periodical allele is dominant. (**a**) The longevity of non-periodical cicada nymphs is 50 years, (**b**) non-periodical cicadas automatically die if they do not mature within a set period (same as Fig. S8a). Mutation rates range from 10^{-3} to 10^{-2} (interval=10^{-3}). **Left**: fixation; **right**: no fixation. Green areas: extinction; orange: fixation of periodicity; blue: no fixation (darker colours indicate higher probabilities). The extinction threshold of the Allee effect is set as N_c=100. The parameters for annual mortality are k=250 and λ=1.005. The penalty of reproduction for immature periodical adults is set to be dependent on the shortage of accumulated temperature. The results are based on 50 simulation runs for each condition.

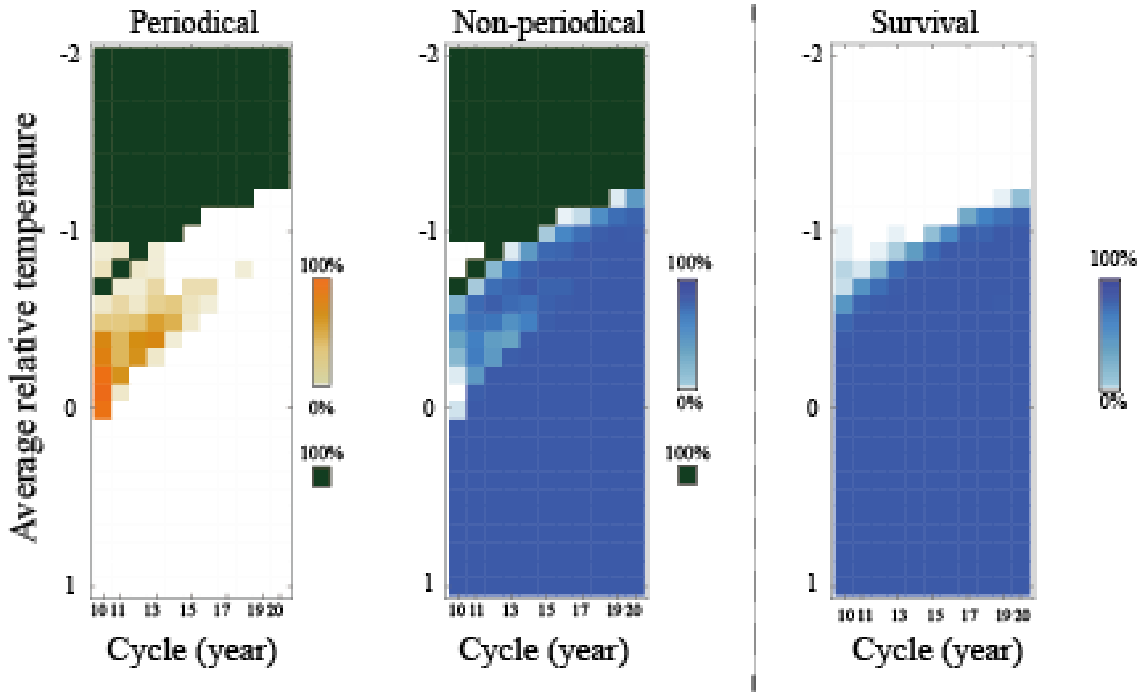

Fig. S11. Phase diagrams of 10- to 20-year periodicity introductions versus average relative temperature (ART) when a single allelic mutation is allowed for an individual. (**left, middle**) Mutation experiment (**left**: fixation; **middle**: no fixation). (**right**) Control (no mutation, survival). Green areas: extinction; orange: fixation of periodicity; blue: no fixation (darker colours indicate higher probabilities). The extinction threshold of the Allee effect is set as N_c=100. The parameters for annual mortality are k=250 and λ=1.005. The penalty of reproduction due to immature periodical cicadas is set to be dependent on the shortage of accumulated temperature. The results are based on 50 simulation runs for each condition. See text for details.

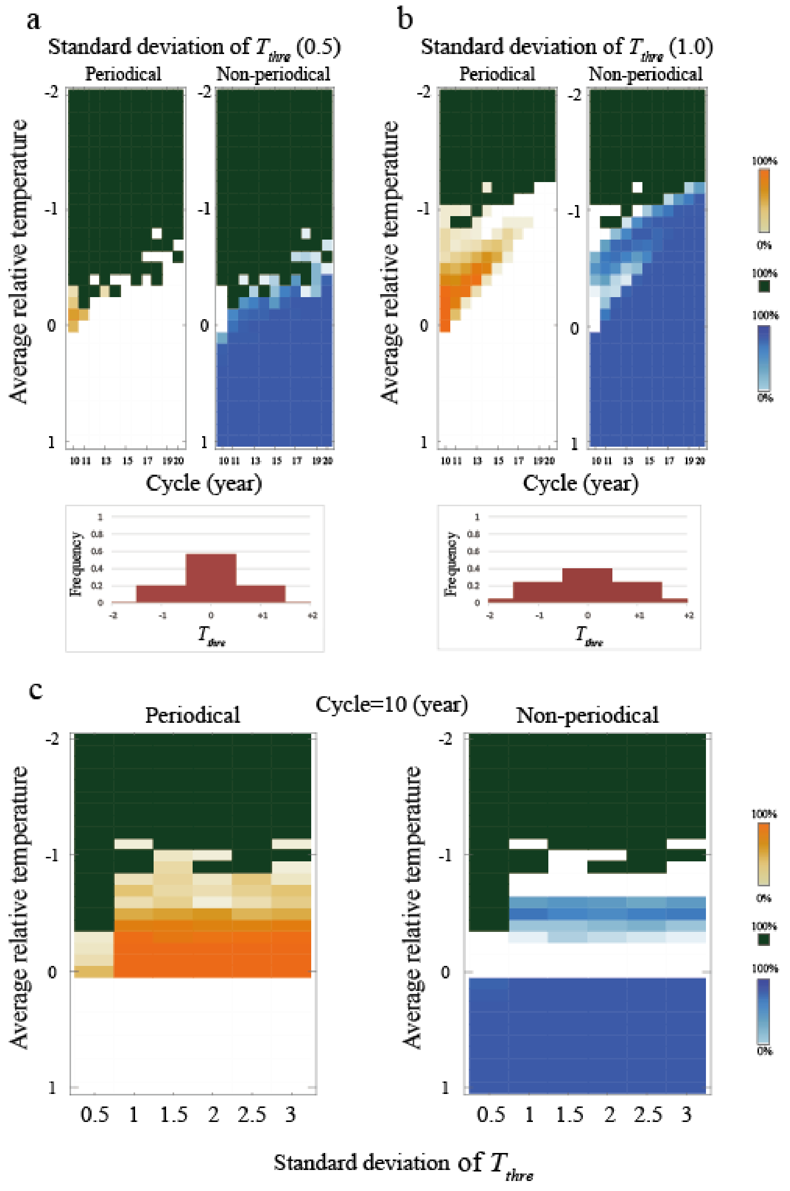

Fig. S12. (a, b) Phase diagrams of 10- to 20-year periodicity introductions versus average relative temperature (ART) with variability in microhabitats. Microhabitat variation follow a discretized and truncated normal distribution. (**a**) Low variability (standard deviation=0.5) and (**b**) high variability (standard deviation=1.0). (**c**) Varying variability (standard deviation=0.5-3.0). (**left**: fixation; **right**: no fixation). Green areas: extinction; orange: fixation of periodicity; blue: no fixation (darker colours indicate higher probabilities). The Allee effect is set as N_c=100. The parameters for annual mortality are k=250 and λ=1.005. The penalty of reproduction for immature periodical adults is set to be dependent on the shortage of accumulated temperature. The results are based on 50 simulation runs for each condition.

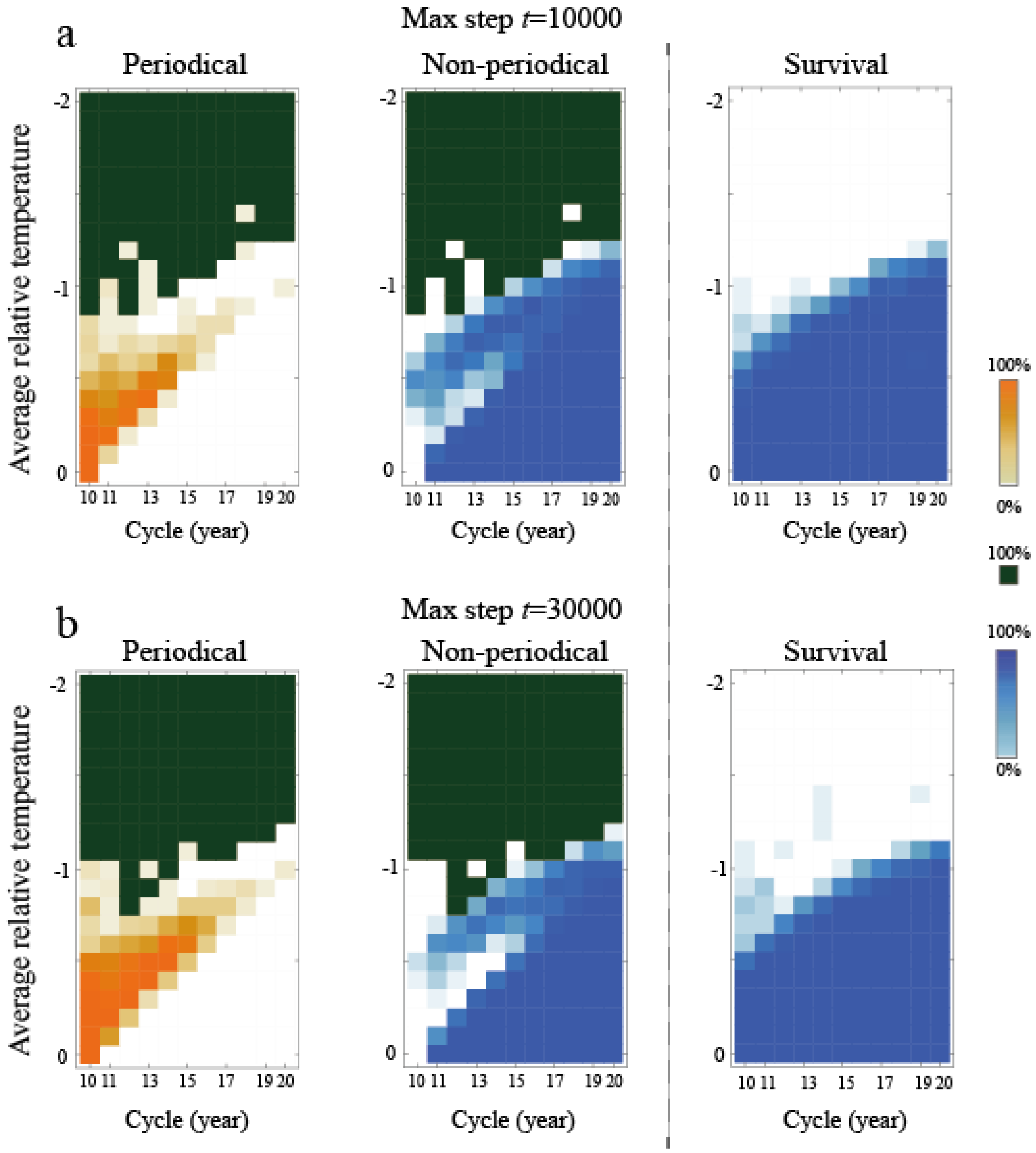

Fig. S13. Phase diagrams of 10- to 20-year periodicity introductions versus average relative temperature (ART) with two different final time steps. (**a**) Final t=10,000, and (**b**) final t=30,000. (**left, middle**) Mutation experiment (**left**: fixation; **middle**: no fixation). (**right**) Control (no mutation, survival). Green areas: extinction; orange: fixation of periodicity; blue: no fixation (darker colours indicate higher probabilities). The extinction threshold of the Allee effect is set as N_c=100. The parameters for annual mortality are k=250 and λ=1.005. The penalty of reproduction for immature periodical adults is set to be dependent on the shortage of accumulated temperature. The results are based on 50 simulation runs for each condition. ART is varied from -2 to 0 because the simulation time is exceedingly elongated if ART>0.

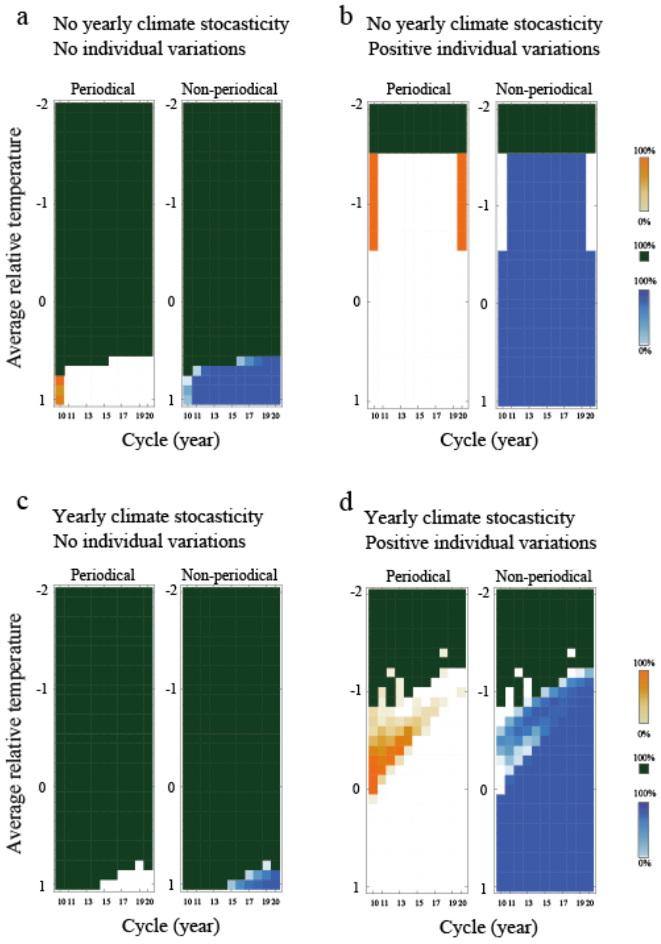

Fig. S14. Phase diagrams of 10- to 20-year periodicity introductions versus average relative temperature (ART) with/without yearly climate stochasticity and individual microhabitat variations. (**a**) No climate stochasticity and no individual variations, (**b**) no climate stochasticity but positive individual variations, (**c**) positive climate stochasticity but no individual variations, and (**d**) positive climate stochasticity and positive individual variations (same as Fig. 3). (**left**) Fixation, (**right)** no fixation. Green areas: extinction; orange: fixation of periodicity; blue: no fixation (darker colours indicate higher probabilities). The extinction threshold of the Allee effect is set as N_c=100. The parameters for annual mortality are k=250 and λ=1.005. The penalty of reproduction for immature periodical adults is set to be dependent on the shortage of accumulated temperature. The results are based on 50 simulation runs for each condition. Note that cycles (periods) correspond with the climatic conditions that evolve with periodicity when there is no yearly climate stochasticity, e.g., during the 10-year period in (**a**) and (**b**). Without individual microhabitat variations (**a** and **c**), the extinction boundary is greatly extended towards the warm climate because all individuals are very similar.
